# Enhanced Surface Plasmon Resonance Wavelength Shifts by Molecular Electronic Absorption in Far- and Deep-Ultraviolet Regions

**DOI:** 10.1038/s41598-020-66949-z

**Published:** 2020-06-18

**Authors:** Ichiro Tanabe, Yoshito Y. Tanaka, Koji Watari, Wataru Inami, Yoshimasa Kawata, Yukihiro Ozaki

**Affiliations:** 1 Graduate School of Engineering Science, Osaka University, Machikaneyama 1-3, Toyonaka, Osaka 6508531 Japan; 2 Institute of Industrial Science, the University of Tokyo, 4-6-1 Komaba, Meguro, Tokyo 1538505 Japan; 3 School of Science and Technology, Kwansei Gakuin University, Gakuen 2-1, Sanda, Hyogo 6691337 Japan; 4 Research Institute of Electronics, Shizuoka University, 3-5-1 Johoku, Hamamatsu, Shizuoka 4328561 Japan

**Keywords:** Chemistry, Analytical chemistry, Photochemistry, Physical chemistry

## Abstract

In this study, surface plasmon resonance (SPR) wavelength shifts due to molecular electronic absorptions in the far-ultraviolet (FUV, < 200 nm) and deep-ultraviolet (DUV, < 300 nm) regions were investigated by attenuated total reflectance (ATR) spectroscopy. Due to the strong absorption in the DUV region, *N,N*-dimethylformamide (DMF) significantly increased the SPR wavelength shift of Al film. On the other hand, no such shift enhancement was observed in the visible region for Au film because DMF does not have absorbance compared to non-absorbing materials such as water and alcohols. The enhanced SPR wavelength shift, caused by the overlap between SPR and molecular resonance wavelengths in FUV-DUV region, is expected to result in high sensitivity for resonant materials.

## Introduction

Recently, surface plasmon resonance (SPR) and localized SPR (LSPR) in the ultraviolet (UV) region have attracted much attention because of higher energy and more abundant electronic transitions in comparison with visible regions^1–14^. For the UV-SPR investigations, Al is a suitable metal because its plasma frequency (2.4 × 10^16^ s^−1^) is higher than the light frequency in the UV regions^15^. The low cost and natural abundance of Al are also its attractive points. For example, fluorescence enhancement and its imaging of biomolecules and cells using Al were reported^4,5^. In the case of surface-enhanced Raman scattering (SERS) in the UV region, the excitation wavelength was down to 229 nm^6–8^. By using the UV light, resonance effect of various molecules such as nucleobases could be utilized. For other instances, photoelectron emission enhancement^9,10^, tip-enhanced Raman scattering (TERS)^11,12^, and photocatalysis enhancement^13,14^ were investigated by using the UV light and Al.

An SPR sensor, which detects refractive index changes near the surface of a metal film, is one of the most important and typical SPR-based applications and is widely used in biochemistry and environmental chemistry^16–20^. Au films are often adopted in commercial SPR sensors, and the Au-based sensors use the lights in the visible region. In contrast, we have recently proposed novel SPR sensors utilizing shorter-wavelength radiation, particularly those in far-ultraviolet (FUV, < 200 nm) and deep-ultraviolet (DUV, < 300 nm) regions^21–23^. While many SPR sensor targets such as proteins and nucleobases have no absorbance in the visible region, they show characteristic and strong absorptions in the FUV-DUV region^24,25^. Some materials such as saccharides absorb light only in the FUV region^26^. Although Al LSPR–based refractive index sensors have been recently reported^27–31^, the sensors worked not in the DUV region but in the longer wavelength region, where target molecules have no absorbance. Thus, there is no report about interactions between SPR and molecular electronic transitions in the refractive index sensing in the DUV region.

We have recently reported that the SPR wavelength of an Al film coated on a sapphire prism remained in the DUV region and that the Al films worked as a refractive index sensor even in liquids^21^. In order to investigate the SPR properties in the FUV-DUV regions, our original attenuated total reflectance (ATR) spectrometer was used. Although the SPR properties of Al films in the FUV region have been studied since the 1970’s^32–34^, their dependence on the refractive index and FUV-DUV-SPR sensor operation have not been described yet. This is because even O_2_ and H_2_O molecules strongly absorbed light in the FUV region and the investigations had to be performed in a high vacuum atmosphere^32–34^. On the other hand, an ATR prism separates the optics part and the sample part^35,36^. While the optics part is purged with dry N_2_ gas, which does not absorb the FUV light, the sample part is exposed to air. Thus, the atmosphere on the Al film can be controlled. Using this technique, SPR wavelengths of Al film on sapphire and quartz prisms were measured. The SPR wavelength shifted to the longer wavelength with the increase of the refractive index *n* on the Al film. Additionally, the effects of Al film thickness were also investigated^22^. However, effects of absorbance of target molecules in the FUV-DUV region was not investigated.

In the present study, the effects of molecular electronic absorptions on SPR sensing were probed by casting liquid samples (1,1,1,3,3,3-hexafluoro-2-propanol (HFIP), water, 2-propanol, 2-butanol, 1-octanol, *N,N*-dimethylformamide (DMF), and HFIP/DMF mixtures) on an Al film deposited on a sapphire prism. Importantly, DMF showed a strong absorption peak at ~200 nm, whereas other analytes did not have absorbance in the utilized wavelength range (170–300 nm). As a result, it was revealed that, due to the strong electronic absorptions of DMF (i.e., anomalous dispersion of the refractive index *n*) near the SPR wavelength, the SPR shift was significantly enhanced compared to the other samples which had no absorbance. Such enhancements were not induced in the Au-based visible-SPR sensor. These results indicate that the selection of a specific light absorption wavelength of the target material allows its selective detection due to the marked changes of *n*, which is the advantage the FUV-DUV-SPR sensor.

## Results and discussion

### Al-SPR wavelength shifts in the presence of liquids without absorbance

**Figure 1:**
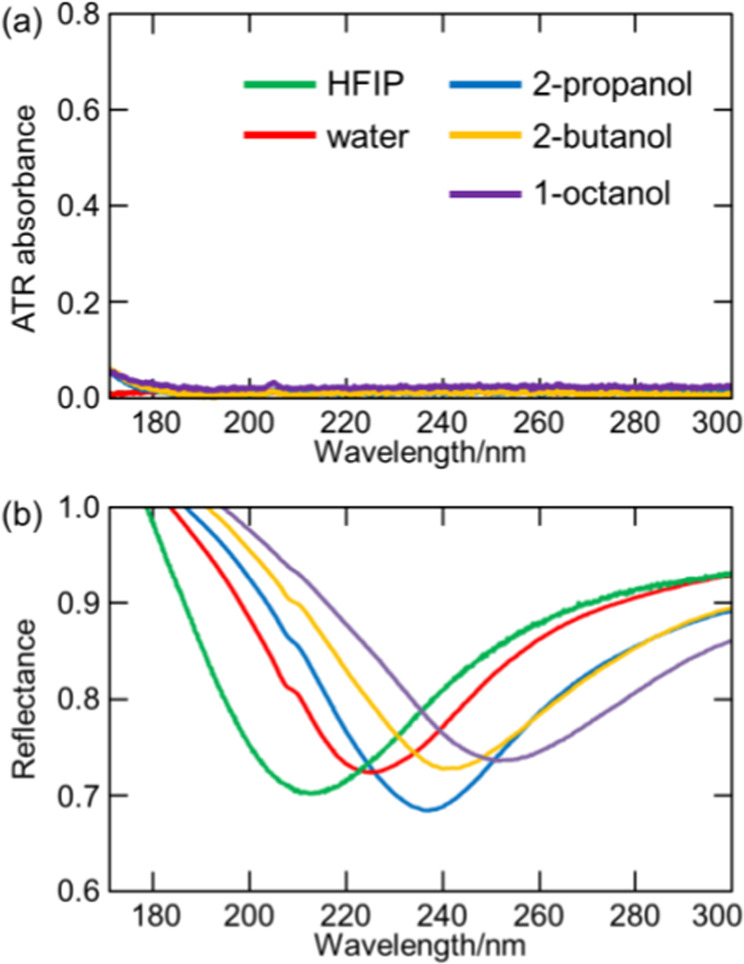
(**a**) Absorption spectra of HFIP, water, 2-propanol, 2-butanol, and 1-octanol. (**b**) Reflection spectra of Al films in the above liquids.

The refractive index *n* of the environment close to the Al film surface was tuned by casting of HFIP, water, 2-propanol, 2-butanol, and 1-octanol one by one (n_D_ = 1.275, 1.333, 1.374, 1.396, and 1.428 at 589.3 nm, respectively). The values of n_D_ present the refractive indices measured at 589.3 nm. ATR absorbance was defined as −log (*I*/*I*_0_), where *I* and *I*_0_ are the reflected light intensities obtained in the presence and absence of a given sample on the sapphire prism, respectively. Figure 1a shows the ATR absorption spectra of these liquid samples. As already mentioned, the above liquids did not have absorbance in the measured wavelength range.

Figure 1b shows the reflection spectra of Al films in HFIP, water, 2-propanol, 2-butanol, and 1-octanol. Reflectance was defined as *I*_sample_/*I*_air_, where *I*_sample_ and *I*_air_ are the intensities of light reflected from the Al film with and without samples, respectively, i.e., the reflectance spectrum measured in air was used as a reference. As discussed previously^23^, the Al film/sapphire prism/air system did not exhibit any SPR absorbance and featured an almost constant reflectance intensity in the 170–300 nm range at an incident angle of 70°. Therefore, the reflection spectrum in air could be used as a reference to determine the SPR wavelengths of Al film on which samples were deposited. The SPR wavelengths of the Al in the presence of HFIP, water, 2-propanol, 2-butanol, and 1-octanol were 215.2, 227.5, 238.8, 242.3, and 253.6 nm, respectively. Notably, the sample refractive index *n* and the SPR wavelength were positively correlated.

The SPR wavelength shift induced by changing of *n* on the Al film was simulated using the Fresnel equation as discussed in Appendix A. The effects of Al film oxidation was also discussed in Appendix A. The minimum reflectance was not equal to zero (~0.7; Fig. 1b), which was ascribed to the fact that whereas the utilized incident light comprises both *p*- and *s*-polarized components, only *p*-polarized light can trigger SPR. However, the space constraints of the present system made it difficult to set a polarizer for the FUV-DUV region.

### Al-SPR wavelength shifts in the presence of liquids with absorbance

**Figure 2:**
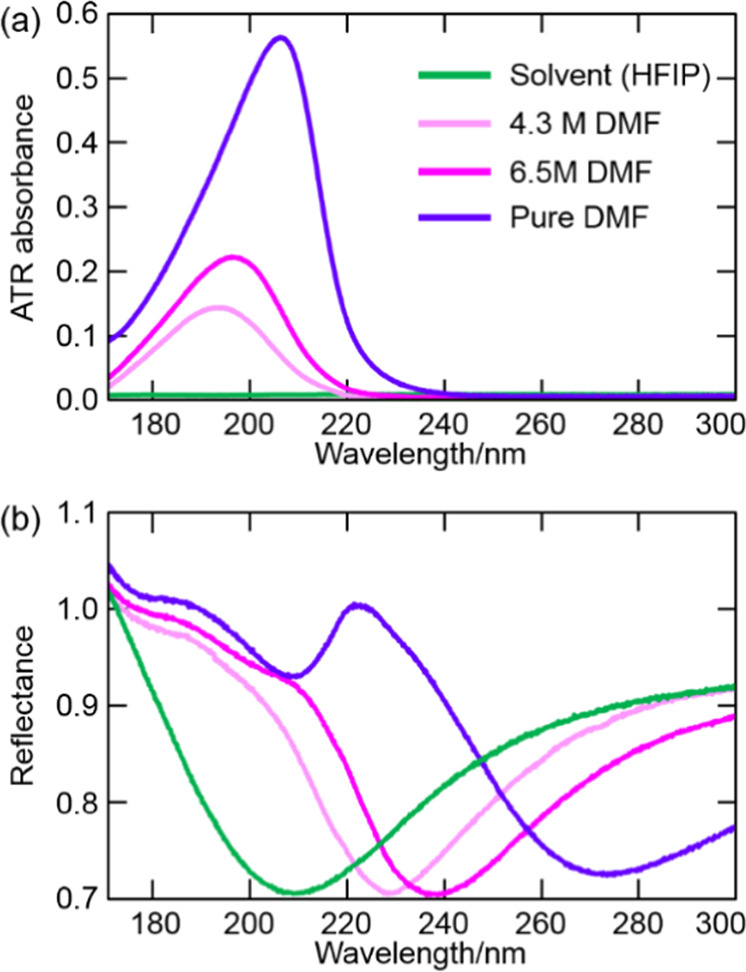
(**a**) ATR absorption spectra of DMF and its mixtures with HFIP. (**b**) Reflection spectra of Al films in the above liquids.

Subsequently, to investigate the effects of material absorbance (i.e., the extinction coefficient *k* and the anomalous dispersion of *n*), DMF and DMF/HFIP mixtures were cast on Al films. The ATR absorbance spectra of HFIP (as solvent), DMF, and DMF/HFIP mixtures are shown in Fig. 2a. In contrast to the liquid samples in Fig. 1a, DMF had a strong absorbance around 200 nm. Because of the influence of the refractive index *n* of the samples, the ATR spectral shapes around the peak wavelength were distorted depending on the DMF concentration. For 4.3 M, 6.5 M, and pure (13.0 M) DMF, the values of n_D_ measured by a refractive index sensor (PAL-RI, Atago Co.) equalled 1.328, 1.350, and 1.429, respectively.

These DMF solutions were put on Al films, and reflection spectra were measured. As shown in Fig. 2b, the SPR wavelength increased with increasing DMF concentration (i.e., with increasing *n*). The SPR wavelengths were 215.2, 233.5, 243.0, and 275.6 nm for HFIP, 4.3 M DMF, 6.5 M DMF, and pure DMF, respectively. The reflection dip due to the absorbance of DMF was also observed around 210 nm in the reflection spectrum using pure DMF (Fig. 2b, purple line), which was not affected by the environmental changes.

**Figure 3:**
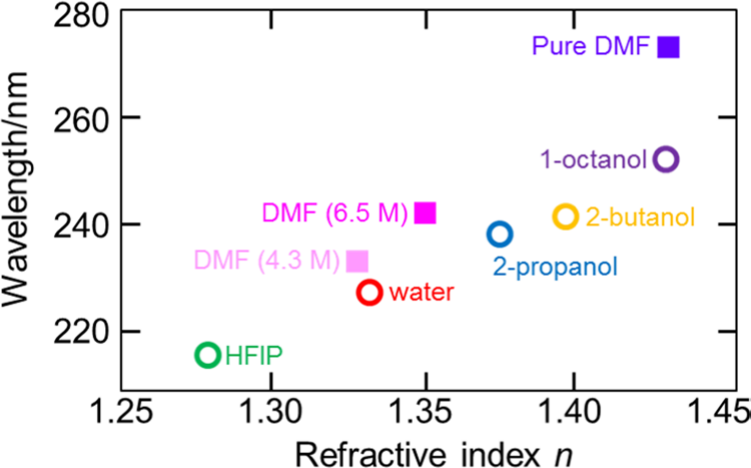
Al-SPR wavelength dependence on the refractive index *n* at 589.3 nm of the samples (open circles) without or (filled squares) with absorbance in the measured wavelength range (170–300 nm).

Figure 3 shows the relationship between *n* and SPR wavelength, with open circles and filled squares being based on the data in Figs. 1b and 2b, respectively.

It should be emphasized here that the refractive index *n* in Fig. 3 was measured at 589.3 nm, which is the wavelength of the sodium D line, and all samples including DMF did not have absorbance in the visible region. As noted above, DMF exhibited a strong absorbance around 200 nm, while other samples had no absorbance in the utilized wavelength range. Since *n* was subject to significant variation around the absorption wavelength (which is denoted as an anomalous dispersion of the refractive index), the use of light with a wavelength close to this value was expected to induce a large SPR wavelength shift. Actually, as shown in Fig. 3, DMF solutions induced a larger shift than water and alcohols, which should result in increased SPR sensor sensitivity. Thus, if the absorption wavelength of DMF is employed, the Al SPR sensor should detect DMF in preference to other materials, illustrating the possibility of using FUV-DUV-SPR sensors for highly sensitive and selective analysis. Further quantitative details regarding SPR sensor performance are provided in the Supporting Information (Appendix B).

### Au-SPR wavelength shifts in the visible region

**Figure 4:**
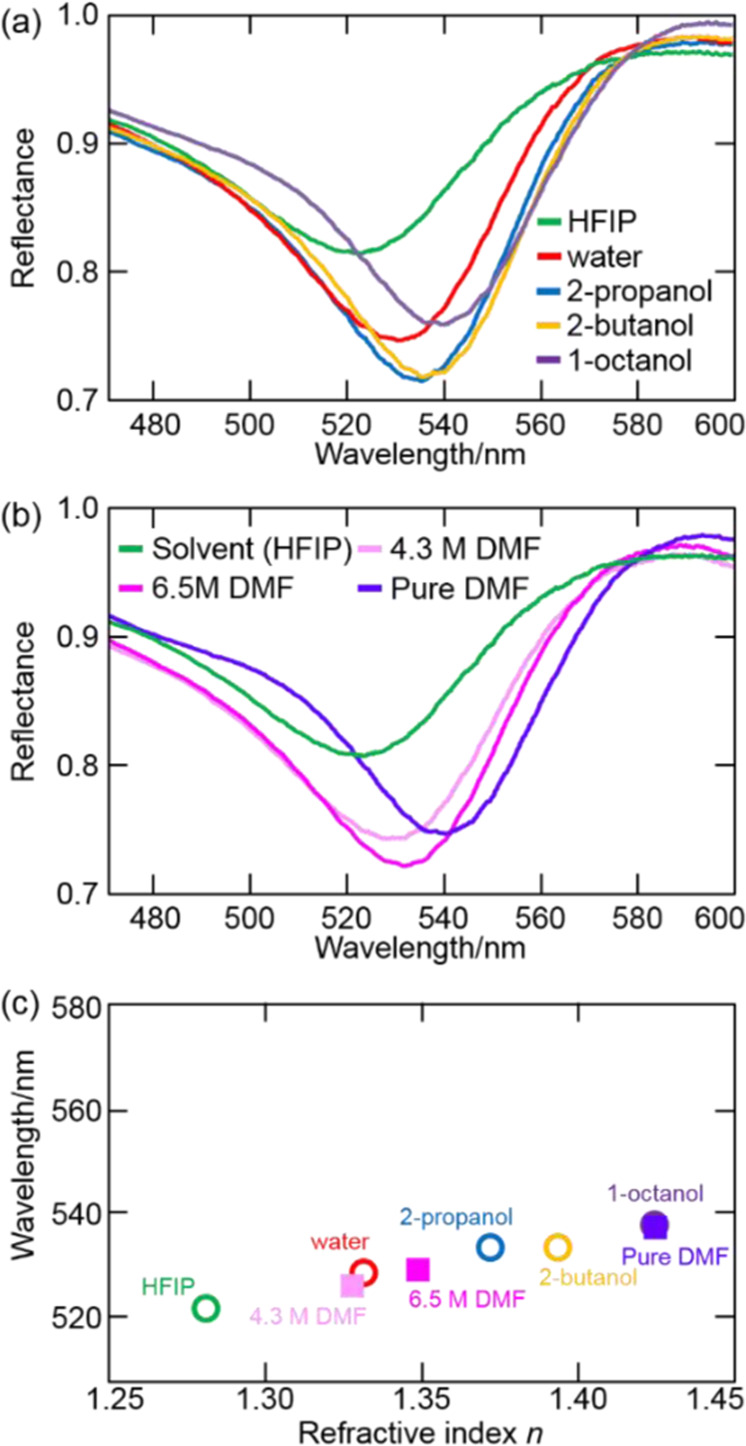
(**a, b**) Au film/sapphire prism reflection spectra in (**a**) HFIP, water, 2-propanol, 2-butanol, 1-octanol, and (**b**) DMF/HFIP mixtures. (**c**) Dependence of Au SPR wavelength on the *n* values (589.3 nm) of different samples.

Next to that, in order to demonstrate the advantages of the developed FUV-DUV-SPR system as a sensor, we compared it with a common Au film/sapphire prism-based SPR sensor operating in the visible region. As ascribed above, the Au-based SPR sensor is widely used in various fields. Figure 4a and b show the obtained reflection spectra, and Fig. 4c summarizes the dependence of the Au SPR wavelength on *n*.

As for the Al film in the FUV-DUV region, *n* was positively correlated with the Au SPR wavelength, which equalled 523.9, 530.9, 536.0, 536.0, 540.6, 528.3, 531.5, and 539.9 nm for HFIP, water, 2-propanol, 2-butanol, 1-octanol, 4.3 M DMF, 6.5 M DMF, and pure DMF, respectively. However, contrary to the case of the Al film, DMF did not induce a marked Au SPR wavelength shift (Fig. 4c) because DMF also had no absorbance (i.e., no rapid change of *n*) in the visible region, similarly to other samples. Further details regarding the performance of the visible-light SPR sensor are provided in the Supporting Information (Appendix C).

## Conclusions

In summary, the effect of the molecular electronic absorption on the SPR wavelength shift was investigated in the FUV-DUV region. Due to the overlap between the SPR wavelength of the Al film and the absorption wavelength of DMF, the SPR shift was enhanced compared to non-resonant materials such as water and alcohols. Such enhancement was not observed for the Au-based visible-SPR sensor because DMF had no absorbance in the visible region. These results indicate that the resonant molecules can be detected sensitively. Many target molecules of the SPR sensor such as nucleotides and proteins have strong absorbance in the FUV-DUV region, and thus, the present FUV-DUV-SPR sensor will lead to the development of novel SPR sensors.

## Methods

The Kretschmann configuration was used to excite the SPR of an Al film deposited on a sapphire prism (purchased from Opto-line, Tokyo) by vapour deposition (5.0 × 10^−4^ Pa, deposition rate ~10 nm s^−1^). The thickness and surface roughness of the above film were determined as 23.0 ± 0.5 nm by atomic force microscopy.

The prepared Al film was set in on the ATR spectrometer, and reflection spectra were recorded at an incident angle of 70° in the range of 170–300 nm. In order to change the environment close to the Al film surface, HFIP, water, 2-propanol, 2-butanol, 1-octanol, DMF, and HFIP/DMF mixtures were cast on the Al film. We performed simulations based on the Fresnel equations using a bilayer (aluminium and alumina) model to analyse the experimentally obtained spectra.

In order to compare the FUV-DUV-SPR sensor with the visible-SPR sensor, SPR wavelengths of Au films in the visible region were investigated. To acquire visible-region reflection spectra using the same instrument as that used for the FUV-DUV region, the light source was replaced from a deuterium lamp to a Xenon lamp, and the diffraction grating was exchanged to that suitable for the visible region. The measured wavelength range was from 470 to 600 nm. Au films with an optimized thickness (~35 nm) were deposited on the sapphire prism (5.0 × 10^−4^ Pa, ~1 nm s^−1^), and the analyte-induced SPR wavelength shifts were investigated.

## Supplementary information

Supplementary Information.
