# Supplementary material for: Enhanced Surface Plasmon Resonance Wavelength Shifts by Molecular Electronic Absorption in Far- and Deep-Ultraviolet Regions
Source: Sci Rep. 2020 Jun 18;10:9938. doi: 10.1038/s41598-020-66949-z (PMC7303190; doi:10.1038/s41598-020-66949-z)
Supplement: Supplementary file 1 — Supplementary Information. [file 41598_2020_66949_MOESM1_ESM.docx]

**Supplementary Information**

**Enhanced Surface Plasmon Resonance Wavelength Shifts by Molecular Electronic Absorption in Far- and Deep-Ultraviolet Regions**

Ichiro Tanabe,^1*^ Yoshito Y. Tanaka,^2*^ Koji Watari,^3^ Wataru Inami,^4^ Yoshimasa Kawata,^4^ and Yukihiro Ozaki^3^

1 Graduate School of Engineering Science, Osaka University, Machikaneyama 1-3, Toyonaka, Osaka 6508531, Japan

2 Institute of Industrial Science, the University of Tokyo, 4-6-1 Komaba, Meguro, Tokyo 1538505, Japan

3 School of Science and Technology, Kwansei Gakuin University, Gakuen 2-1, Sanda, Hyogo 6691337, Japan

4 Research Institute of Electronics, Shizuoka University, 3-5-1 Johoku, Hamamatsu, Shizuoka 4328561, Japan

**APPENDIX A: Consistency between experimental and simulated results, and the effects of Al film oxidation.**


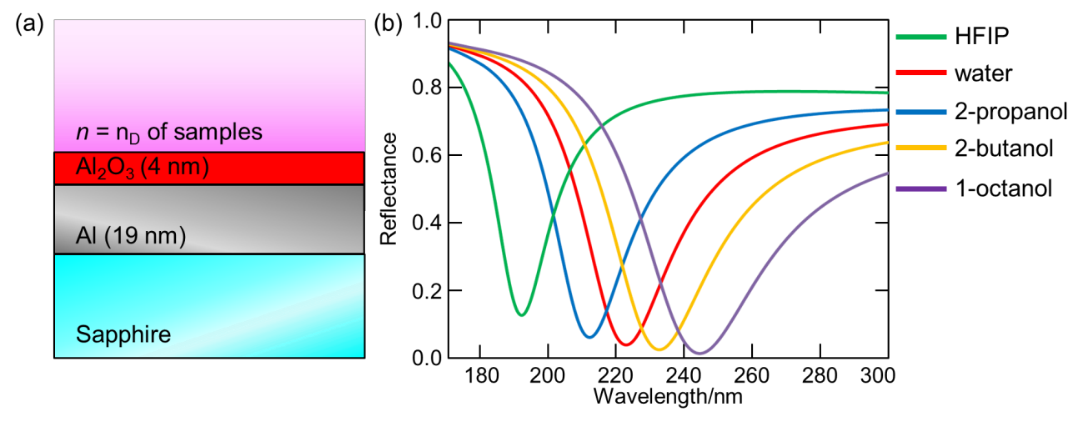


**Figure S1.** (a) A simulation model and (b) simulated reflection spectra obtained using the Fresnel equation.

A bilayer (Al 19 nm, Al_2_O_3_ 4 nm) model was adopted in the present simulation. In a previous report,^1^ the thickness of the Al_2_O_3_ layer in the simulation model was changed from 0 to 6 nm, and the best agreement with experimental data was observed for a thickness of 4 nm. The simulated SPR wavelengths obtained herein equaled 191, 211, 222, 232, and 244 nm for bilayer film refractive indices *n* of 1.275 (HFIP), 1.333 (water), 1.374 (2-propanol), 1.396 (2-butanol), and 1.428 (1-octanol), respectively. The experimentally obtained SPR wavelengths were exceeded the simulated ones, equaling 215.2, 227.5, 238.8, 242.3, and 253.6 nm for HFIP, water, 2-propanol, 2-butanol, and 1-octanol, respectively, since the *n* values in the DUV region exceeded those obtained by simulation (*n* at 589.3 nm). Notably, when we used the HFIP refractive indices in the FUV and DUV regions calculated by density functional theory (functional: M062X; basis set: aug-cc-pVTZ) with Gaussian 09 (Rev C.01),^2^ a better match as observed.^1^

It should also be noted that the thickness of naturally formed Al_2_O_3_ films on Al single crystals equaled ~3 nm,^3^ i.e., a 4-nm Al_2_O_3_ layer was overly thick. This inconsistency is probably due to the differences between modeled and measured films. Although a complete bilayer model of Al and Al_2_O_3_ was adopted in the simulation, the oxidation of Al should occur even in the Al film, e.g., the oxidation of Al was reported to occur even at a low pressure (~4 × 10^−5^ Pa) of oxygen.^4^ Moreover, SPR is also affected by the surface roughness of the metal film.^5^

Thus, we emphasized the advantage of the presence of an oxidation layer. The growth of naturally formed aluminum oxide films has been reported to stop upon reaching a thickness of about 3 nm,^3^ which means that the Al film can be used as a stable sensor after sufficient oxidation, as demonstrated in our previous paper.^6^

(1) Tanabe, I.; Tanaka, Y. Y.; Ryoki, T.; Watari, K.; Goto, T.; Kikawada, M.; Inami, W.; Kawata, Y.; Ozaki, Y. *Opt. Express* **2016,** *24*, 21886–21896.

(2) Frisch, M. J.; Trucks, G. W.; Schlegel, H. B.; Scuseria, G. E.; Robb, M. A.; Cheeseman, J. R.; Scalmani, G.; Barone, V.; Mennucci, B.; Petersson, G. A.; Nakatsuji, H.; Caricato, M.; Li, X.; Hratchian, H. P.; Izmaylov, A. F.; Bloino, J.; Zheng, G.; Sonnenberg, J. L.; Hada, M.; Ehara, M.; Toyota, K.; Fukuda, R.; Hasegawa, J.; Ishida, M.; Nakajima, T.; Honda, Y.; Kitao, O.; Nakai, H.; Vreven, T.; Montgomery, J. A., Jr.; Peralta, J. E.; Ogliaro, F.; Bearpark, M.; Heyd, J. J.; Brothers, E.; Kudin, K. N.; Staroverov, V. N.; Kobayashi, R.; Normand, J.; Raghavachari, K.; Rendell, A.; Burant, J. C.; Iyengar, S. S.; Tomasi, J.; Cossi, M.; Rega, N.; Millam, J. M.; Klene, M.; Knox, J. E.; Cross, J. B.; Bakken, V.; Adamo, C.; Jaramillo, J.; Gomperts, R.; Stratmann, R. E.; Yazyev, O.; Austin, A. J.; Cammi, R.; Pomelli, C.; Ochterski, J. W.; Martin, R. L.; Morokuma, K.; Zakrzewski, V. G.; Voth, G. A.; Salvador, P.; Dannenberg, J. J.; Dapprich, S.; Daniels, A. D.; Farkas, O.; Foresman, J. B.; Ortiz, J. V.; Cioslowski, J.; Fox, D. J. *Gaussian 09*, revision B.01; Gaussian, Inc.: Wallingford, CT, 2009.

(3) Doherty, P. E.; Davis, R. S. *J. Appl. Phys.* **1963**, *34*, 619–628.

(4) Kirk, C. T. Jr.; Huber, E. E. Jr. *Surf. Sci.* **1968**, *9*, 217–245.

(5) Endriz, J. G.; Spicer, W. E. *Phys. Rev. Lett.***1970**, *24*, 64–68.

(6) Tanabe, I.; Tanaka, Y. Y.; Watari, K.; Hanulia, T.; Goto, T.; Inami, W.; Kawata, Y.; Ozaki, Y. *Sci. Rep.* **2017,** *7*, 5934.

**APPENDIX B: SPR sensing ability of an Al film in the FUV-DUV region.**

As shown in Figure 3, even though pure DMF (1.429) and 1-octanol (1.428) exhibited almost identical *n* values in the visible region (589.3 nm), the SPR wavelength shift induced by pure DMF (60.4 nm) exceeded that induced by 1-octanol (38.4 nm).

From a practical viewpoint, the change of reflected light intensity at a fixed incident angle and wavelength is important. In the present case, the incident angle was fixed at 70°. Since DMF featured an absorbance peak at ~200 nm, we focused on relative reflection intensity at 200 nm, with that of HFIP equaling ~0.79 (Figures 1a and 2a). The reflection intensity changed to ~0.91 in the presence of water, further increasing to ~0.96 in the presence of 4.3 M DMF. Even though the visible-region (589.3 nm) *n* of 4.3 M DMF (1.328) was smaller than that of water (1.333), the former system induced a larger reflected light intensity change.

**APPENDIX C: SPR sensing abilities of Al and Au films in the visible region.**

Although Al film can be used as an SPR sensor both in the UV and visible regions, the large imaginary part of its dielectric constant in the latter region is not suitable for SPR sensor operation.

It should be noted that the results shown in Figure 4 were obtained using a Au film on a sapphire prism. However, quartz prisms are often used for Au-based visible-region SPR sensors due to affording larger SPR wavelength shifts than those obtained using sapphire prisms.2a Although a 70° incident angle was used herein, this value should be further optimized for practical applications. Therefore, although the SPR wavelength shift of the Al film (Figure 3) exceeded that of the Au film (Figure 4c) in the present study, this should not always be the case. Thus, we demonstrated the ability of the FUV-DUV-SPR sensor to selectively detect certain molecules, with the further advantages of using FUV and DUV regions being increased sensitivity and material selectivity.
